# Comparative Evaluation of the Fracture Resistance of Two Different Fiber-reinforced Composite Restorations with Particulate Filler Composite Restorations

**DOI:** 10.5005/jp-journals-10005-1526

**Published:** 2018-08-01

**Authors:** Arun K Patnana, Vanga V Narasimha Rao, Srinivas K Chandrabhatla, Vabbala R Rajasekhar

**Affiliations:** 1 Resident Doctor, Department of Dentistry, All India Institute of Medical Sciences Jodhpur, Rajasthan, India; 2 Professor, Department of Pedodontics and Preventive Dentistry, GITAM Dental College & Hospital, Visakhapatnam, Andhra Pradesh India; 3 Professor, Department of Pedodontics and Preventive Dentistry, GITAM Dental College & Hospital, Visakhapatnam, Andhra Pradesh India; 4 Professor, Department of Pedodontics and Preventive Dentistry, GITAM Dental College & Hospital, Visakhapatnam, Andhra Pradesh India

**Keywords:** Fracture resistance, Glass fiber-reinforced composites, Incisal fractures, Mesio-incisal fractures, Polyethylene fiber-reinforced composites.

## Abstract

Uncomplicated crown fractures are the most common form of traumatic dental injuries (TDIs) in children affecting their personal and social well-being.

**Aim:**

To evaluate the fracture resistance of fractured incisors restored with particulate filler composites, glass fiber-reinforced composites, and Polyethylene fiber-reinforced composite restorations.

**Materials and methods:**

Standardized incisal and mesio-incisal fractures with chamfer preparation were prepared on human maxillary central incisors. Test samples were restored using particulate filler composites (Filtek Z 250), glass fiber-reinforced composites (Fiber-Splint) and polyethylene-reinforced composites (Ribbond). Static load was applied to the test samples using Universal testing machine at a cross-head speed of 1 mm/min. Data were tabulated and analyzed using analysis of variance (ANOVA) (p = 0.05).

**Results:**

Descriptive statistics of mean [standard deviation (SD)] peak failure load in incisal restorations for particular filler composite, glass fiber-reinforced composites, and polyethylene fiber-reinforced composites were 196.00 (± 67.46), 186.28 (± 66.44), and 246.71 (± 24.52) respectively, whereas for mesio-incisal restorations, mean (SD) peak failure loads were 169.28 (± 33.53), 218.57 (± 74.41), and 225.71 (± 57.52) respectively.

**Conclusion:**

Polyethylene-reinforced composites showed an improved load-bearing capacity in incisal and mesio-incisal restorations when compared with particulate filler composites and glass fiber-reinforced composites.

**How to cite this article:** Patnana AK, Rao VVN, Chandrabhatla SK, Rajasekhar VR. Comparative Evaluation of the Fracture Resistance of Two Different Fiber-reinforced Composite Restorations with Particulate Filler Composite Restorations. Int J Clin Pediatr Dent 2018;11(4):277-282.

## INTRODUCTION

Anterior crown fractures are a common form of TDIs in children and adolescents.^1^ It is also hypothesized that the incidence of TDI in the future might exceed the incidence of dental caries and periodontal diseases.^2^ The most common TDI among these are the uncomplicated crown fractures, which represent up to 51% of all TDI.^1^ The TDI involving the anterior teeth not only may lead to compromised tooth functioning, speech, and facial esthetics, but may also have an impact on a personality of the child and quality of life.^3^ Hence, immediate treatment of such a condition is required.

The treatment of an uncomplicated coronal fracture is an important challenge for the dentist because many parameters are involved in the successful outcome of the restoration. Over the years, a large number of techniques have been employed for restoration of uncomplicated crown fractures which include stainless steel crowns, orthodontic bands, resin held by pins,^4^ and porcelain crowns.^5^ However, the compromised esthetic outcomes and substantial sacrifice of the tooth structure limit their use in anterior restorations.^6^ Reattachment of the fractured segment is proposed to be a valid alternative for anterior restorations. Though this technique is esthetically acceptable, debonding or refracture of restored segment to the new trauma is the main drawback.^7,8^

In order to withstand the impact forces during retrauma conditions, the ideal restorative material should have high fracture resistance values.^1^ In the past, attempts have been made to improve the load-bearing capacity of restoration by using different bonding systems and adhesive resins.^5,8^ These techniques have reported fracture resistance of 50 to 60% when compared with intact incisors.^5^

In the quest to improve the fracture resistance of the incisal restorations, different types of fibers, such as carbon fibers, Kevlar fibers, Vectran fibers, glass fibers, and polyethylene fibers were incorporated into the resin matrix of composites, which in turn increase the physical and mechanical properties of the restoration. Polyethylene and glass fibers improve the impact strength, modulus of elasticity, and flexural strength of composite materials. Unlike carbon and Kevlar fibers, polyethylene and glass fibers are almost invisible in resinous matrix and for these reasons, polyethylene and glass fibers seem to be the most appropriate and esthetic strengtheners of composite materials in anterior restorations.^9^ However, there is limited literature testing the superiority and strength of the two materials.

Hence, the objective was to assess the static load-bearing capacity of fractured incisal and mesio-incisal edges restored with conventional particulate filler composites, glass fiber-reinforced composites, and polyethylene fiber-reinforced composites.

## MATERIALS AND METHODS

The study was carried out in the Department of Pedo-dontics and Preventive Dentistry, GITAM Dental College & Hospital, Visakhapatnam, India. Human noncarious permanent maxillary central incisors extracted for peri-odontal problems were collected. Teeth with any fracture or craze lines, teeth with incomplete root formation, and teeth with attrition involving incisal edge were excluded from the study. The surface debridement of teeth was done with hand scalers to remove soft tissues and calculus. The test samples were randomly divided into two groups as shown in Table 1.

**Fig. 1:**
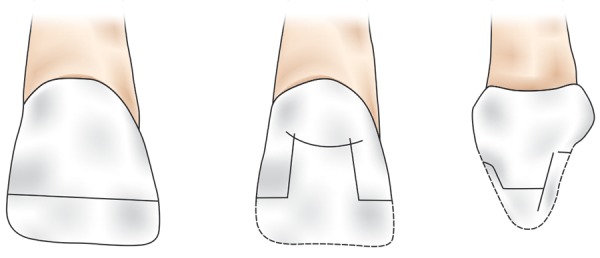
Restorative procedure in incisal fracture group

**Fig. 2:**
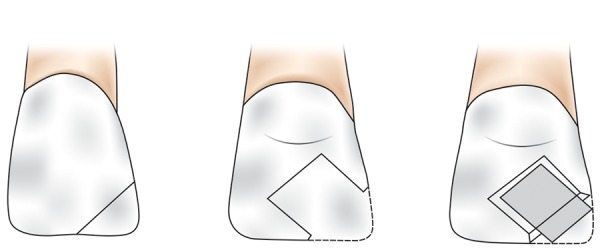
Restorative procedure in mesio-incisal fracture group

Before sample preparations, custom-made strip crown preparation was done for all the samples to achieve original tooth morphology after restoration.^10^ In both the groups, standardized incisal and mesio-incisal fractures were created using diamond disk under water cooling (Figs 1 and 2). A circumferential chamfer was prepared around the sectioned tooth extending 2 mm below the fracture line. In both the groups, the test samples were further subdivided into three subgroups based on the restorative material used as shown in Table 2.

### Restoration with Particulate Filler Composites

Following incisal and mesio-incisal preparations, acid etching (Meta Etchant, Meta Biomed Co. Ltd.) was done and bonding agent (3M Single bond 2) was applied and light cured according to the manufacturer’s instructions. Particulate filler composite (Filtek Z 250 XT, 3M ESPE) was built up and polymerized using handheld light-curing unit. Normal tooth anatomy was restored using custom-made templates for each tooth.

**Table 1:** **Table 1:** Distribution of test samples

*Group*		*Fracture pattern*	
I		Teeth subjected to crown fracture involving incisal edge	
II		Teeth subjected to crown fracture involving mesio-incisal edge	

**Table 2:** **Table 2:** Distribution of test samples in incisal and mesio-incisal restoration groups

		*Subgroup A*		*Subgroup B*		*Subgroup C*	
Incisal restorations (group I) and mesio-incisal restorations (group II)		Particulate filler composite restorations		Glass fiberreinforced composite restorations		Polyethylene fiber-reinforced composite restorations	

### Restoration with Fiber-reinforced Composites

Following the incisal and mesio-incisal preparations, additional cavity preparation (0.5 mm depth, 4 mm mesiodistal width, and 4 mm cervico-incisal height) on the palatal surface of each tooth was done using a diamond bur under water coolant. Following etching, the bonding agent was applied over the fractured tooth surface and in palatal cavity. Required length of the glass and polyethylene fibers (Fiber-Splint, Polydentia SA, Switzerland; Ribbond, Ribbond INC, Seattle, Washington, USA) was measured such that the fiber bundle extended 2 mm below the fracture line.

The fiber was saturated with the bonding agent and excess of the bonding agent was cleared using a gentle air blow. A thin layer of a nanohybrid restorative material (Filtek Z 250, 3M ESPE, St Paul, Minnesota, USA) was carried in the palatal cavity; this thin layer of composite acts as glue and will hold the ribbon during its adaptation. Now, the fiber bundle was placed over composite layer in the palatal cavity such that the fiber extended 2 mm beyond the fracture line and polymerized. Normal tooth anatomy was restored with particulate filler composites using the custom-made templates for each specimen.

**Figs 3A and B:**
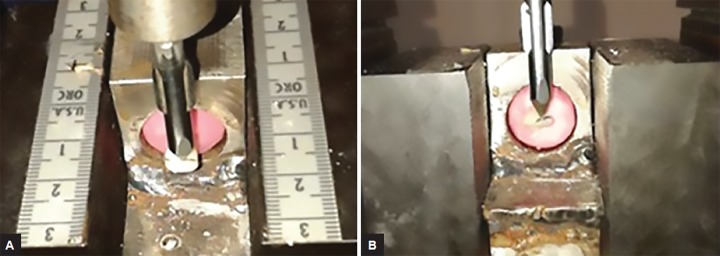
Fracture load application in incisal and mesio-incisal test samples

After completing the restoration in all the groups, samples were stored in distilled water at room temperature for 24 hours before testing. The test samples were mounted in acrylic blocks up to cementoenamel junction using autopolymerized acrylic resin with long axis perpendicular to the base of the block. The acrylic block containing the restored tooth was tightly fixed to the custom-made inclined metal base to provide a 90° angle to the horizontal plane (Fig. 3), which was held on the Universal testing machine (capacity 250 KN and Instron make).

Compressive fatigue load was applied with a loading tip of 1 mm cross-head diameter, at a speed of 1 mm per minute, between the junction of tooth and restoration interface from labial surface with a relative angle of 90° until fracture occurred.^11^ During the testing procedure, a reading of applied load was observed both graphically and numerically. Sudden drop in the load value of graph was considered as peak fracture load in Newton for the particular specimen.

### Statistical Analysis

The data were statistically analyzed using the Statistical Package for the Social Sciences version 20 software. Descriptive statistics were done to calculate the mean, mean difference, SD. Kruskal-Wallis rank test was used to evaluate the significant difference among the three subgroups of each experimental group. When the p-value was less than 0.05, the results were considered statistically significant.

**Table 3:** **Table 3:** Intercomparison of mean peak failure loads for incisal restorations (group I)

*Groups*		*Minimum load value*		*Maximum load value*		*Mean ±SD*		*Mean rank values*		*Chi square values*		*p-value*	
Particulate filler composite		53.00		263.00		196.00 ± 67.46		9.07		4.528		0.104	
Glass fiber-reinforced composite		103.00		273.00		186.28 ± 66.43		8.86					
Polyethylene-reinforced composite		205.00		282.00		246.71 ± 24.52		15.07					

**Table 4:** **Table 4:** Intercomparison of mean peak failure loads for mesio-incisal restorations (group II)

*Groups*		*Minimum load value*		*Maximum load value*		*Mean ±SD*		*Mean rank values*		*Chi square values*		*Significance*	
Particulate filler composite		119		212		169.28 ± 33.53		6.71		5.039		0.081	
Glass fiber-reinforced composite		86		301		218.57 ± 74.41		13.43					
Polyethylene-reinforced composite		153		324		225.71 ± 57.52		12.86					

## RESULTS

**Graph 1:**
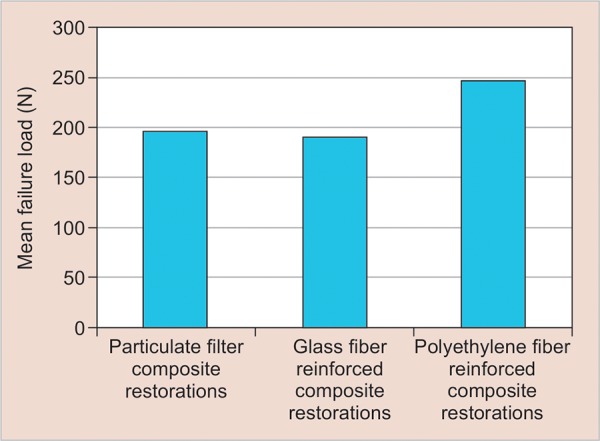
Mean failure loads in incisal restorations

**Graph 2:**
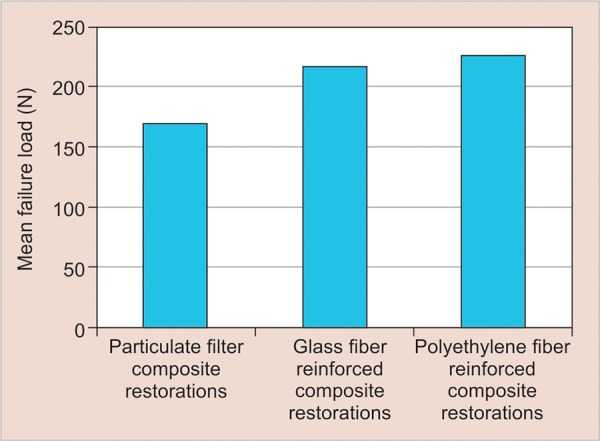
Mean failure loads in mesio-incisal restorations

The mean fracture resistance and SD of incisal and mesio-incisal restoration groups are shown in Tables 3 and 4. The mean fracture loads in incisal and mesio-incisal restoration groups were presented in Graphs 1 and 2 respectively. Data from incisal restorations revealed that highest fracture resistance values were seen in polyethylene fiber-reinforced composites (246.71 ± 24.52) followed by particulate filler composites (196.00 ± 67.46) and glass fiber-reinforced composites (186.28 ± 66.44). In mesio-incisal restorations, highest fracture resistance values were observed in polyethylene fiber-reinforced composites (225.71 ± 57.52) followed by glass fiber-reinforced composites (218.57 ± 74.41) and particulate filler composite restorations (169.28 ± 33.53). However, Kruskal-Wallis rank test showed no statistically significant difference in the mean fracture load values in the three subgroups of incisal (p = 0.104) and mesio-incisal (p = 0.081) restorations.

## DISCUSSION

Extracted human maxillary central incisors were used as the test samples in the present study, as most of the prevalence studies^12-15^ have reported that they are involved in uncomplicated fractures resulting from direct trauma because of its position and protrusion taken during the eruptive process.^2^

In the present study, incisal and mesio-incisal fractures were prepared to test the fracture resistance of fiber-reinforced composites, which was according to different *in vitro* studies^16,19^ for evaluating fracture toughness of composite restorations, re-attachment techniques, and different beveling techniques. Bulk pack technique using custom-made strip crowns was employed for the restorations in order to restore teeth back to their original morphology, thereby reducing the error in standardizing the amount of restorative material used.^10^

All teeth were sectioned at an equal distance from the incisor margin (3 mm) in order to obtain a standardized area of exposure. The anatomy of the surface produced by sectioning is certainly different from the surface resulting from the fracture,^20^ but the choice of sectioning the teeth was dictated by the fact that sectioning establishes a repeatable condition absolutely necessary for an *in vitro* study. A chamfer preparation of 2 mm below the fracture line was prepared on all the test samples in the present study as it improves the mechanical and retentive properties of composite restorations.^16,17,21,22^

Recently, nanocomposites showed distinct mechanical and physical properties compared with conventional resin bonded composites.^23,24^ Owing to its improved mechanical properties and successful clinical outcomes, nanohybrid composites were used as the control restorative material. Incorporation of fibers into the restorative materials has been suggested to increase the fracture resistance of composites. Glass and polyethylene fibers offer better esthetics, impact strength, modulus of elasticity, and flexural strength^25-27^; hence, they were used for reinforcing the composites in the present study.

According to Ellakwa et al,^28^ maximum reinforcing effect of fiber addition is gained by placement of fibers at the tensile side. However, it was also reported that placing the fibers directly on the palatal side may lead to exposure of fibers to the oral environment and may provide a plaque retention factor which in turn lead to premature failure of the restoration.^28^ Thus, in the present study, a thin layer of composite was placed over the fibers to prevent direct exposure to the oral environment.

Addition of fibers along with overlying composites on the palatal surfaces may result in occlusal problems. To overcome this factor, a 0.5 mm of tooth preparation was advised to accommodate the fibers along with overlying composites on the palatal surface.^26^ In accordance with different clinical case reports,^29,30^ a cavity of 0.5 mm depth on the palatal surface was prepared which extends 4 mm mesiodistally and 4 mm occluso-gingivally for all the test samples in the present study.

Successful clinical case reports were published which showed reinforcement of the composites by extending a 2 mm fiber material into the restoration.^21,31^ Thus, in the present study, required length of the fiber material was taken in such a way that it extended 2 mm beyond the fracture line to reinforce the particulate filer composite. According to different *in vitro* studies,^32,33^ loading force was applied perpendicular to long axis of the mounted specimen from labiolingual direction at a constant cross-head speed of 1 mm/min using Universal testing machine.^11^

In both incisal and mesio-incisal restorations, polyethylene fiber-reinforced composites showed a maximum mean peak failure load values when compared with other experimental groups. The results of improved fracture resistance values for fiber-reinforced composites in the present study are in accordance with the clinical case reports and laboratory studies which reported that the fiber acts as individual crack-stopping units.^34^

According to Tezvergil et al^35^ and Garoushi et al,^36^ adequate bonding between the fiber bundle and composite matrix is having critical importance. Polyethylene fibers (Ribbond) shows a semi interpenetrating polymer network (IPN) bonding between the fiber and composite resin matrix, whereas such bonding was absent in the glass fiber-reinforced composites (Fiber-Splint); thus, improved load-bearing values were observed in the polyethylene fiber-reinforced composites than glass fiber-reinforced composite restorations in the present study.

Contrary to the study results of Garoushi et al,^37^ particulate filler composite restorations showed higher fracture resistance values than glass fiber-reinforced composite restorations for incisal restorations in the present study; this might be because of absence of semi IPN bonding in the glass fibers (Fiber-Splint) which in turn allows the propagation of cracks between fibers and composite resin matrix and resulted in decreased load-bearing values.

However, in mesio-incisal restorations, glass fiber-reinforced composites showed higher fracture load values when compared with particulate filler composites alone. These findings are in accordance with the study results of Vallittu^38^ where the concept of total fiber reinforcement *vs* partial fiber reinforcement was discussed and inferred that the reinforcing ability of the fibers will improve by increasing the area of fiber in the dentures. Similarly, in the present study, there is an increased proportion in the area of glass fibers over fractured tooth surface for mesio-incisal restorations, which resulted in improved fracture resistance values than for incisal restorations.

## CONCLUSION

Analyzing the fracture resistance values in the three experimental groups, it can be concluded that polyethylene fibers (Ribbond) efficiently reinforces the incisal and mesio-incisal restorations by imparting higher stiffness to the tooth structure which in turn increases the load-bearing capacity of the tooth restoration complex.

Though every effort was taken to duplicate the oral situations in the present study, *in vivo* responses to the direction and impact forces might differ from the current results. The results of this *in vitro* investigation must be extrapolated to the clinical situation with care and further *in vivo* trials with these materials are indicated to confirm the validity of these recommendations.
